# Social determinants of health inequalities in early phase clinical trials in Northern England

**DOI:** 10.1038/s41416-024-02765-w

**Published:** 2024-06-24

**Authors:** S. Rae, S. Shaya, E. Taylor, J. Hoben, D. Oluwashegun, H. Lowe, N. Haris, S. Bashir, C. Oing, M. G. Krebs, F. C. Thistlethwaite, L. Carter, N. Cook, A. Greystoke, D. M. Graham, R. Plummer

**Affiliations:** 1 Sir Bobby Robson Cancer Trials Research Centre, Northern Centre for Cancer Care, Freeman Hospital, Freeman Road, Newcastle upon Tyne, NE7 7DN UK; 2 Newcastle University, Newcastle upon Tyne, NE1 7RU UK; 3 The Christie NHS Foundation Trust, Wilmslow Road, Manchester, M20 4BX UK; 4 University of Manchester, Oxford Road, Manchester, M13 9PL UK

**Keywords:** Cancer therapy, Clinical trials

## Abstract

**Background:**

Early phase clinical trials in Oncology represent a subspecialised area where UK patient selection is influenced by access to Experimental Cancer Medicine Centres (ECMCs). Equity of access with respect to social determinants of health (SDoH) were explored for two major ECMCs.

**Methods:**

A retrospective cohort study including all referrals to Newcastle and Manchester ECMCs in 2021 was completed. Consent to screening or pre-screening was stratified against SDoH characteristics, including: Index of Multiple Deprivation (IMD) decile, ethnicity and distance to centre.

**Results:**

1243 patients were referred for trials. IMD quintile 1 (most deprived) patients had reduced likelihood of referral compared to expected population models (OR, 0.67; 95% CI: 0.55 to 0.80, *p* = <0.0001). IMD quintile 5 (least deprived) had increased likelihood of referral (OR, 1.46; 95% CI: 1.17 to 1.82, *p* = 0.0007). Living beyond median distance from Manchester reduced the likelihood of consenting to trials (OR, 0.72; 95% CI: 0.55 to 0.94, *p* = 0.015). Ethnicity data represented a White British propensity.

**Conclusions:**

Inequalities in socioeconomic and geographic factors influence referral and enrolment to early phase clinical trials in Northern England. This has implications for equity of access and generalisability of trial results internationally and warrants further study.

## Introduction

There is a growing recognition that social determinants of health, and their resulting inequalities, impact patients with cancer throughout their disease course [1, 2]. These determinants of healthcare inequalities can be characterised as *“avoidable, unfair and systemic differences in health between different groups of people”* which across a patient’s life impacts on their health and the medical care which is accessible to them [3] Such disparities, and how we address them in cancer research, has been established as a priority by both the NHS and CRUK [4, 5].

Early phase trials in oncology offer patients with advanced cancer access to experimental therapies. The majority of these are conducted in the UK through the Experimental Cancer Medicine Centres (ECMCs) network [6]. This represents a highly specialised area of cancer research, where suitable patients are carefully selected for trials to maximise the safety of participants and the tolerability of experimental medicines. It has been found that patients are disproportionately recruited from white ethnic and higher socioeconomic status populations [7, 8]. Representative populations in clinical trials are key to establishing efficacy of drugs across patient groups as well as identifying significant differences in response and tolerability to educate future trial design [9]. Establishing equity of access to early phase clinical trials should be prioritised to improve upon the present cancer outcome disparities.

Cancer does not impact people equally from the point of diagnosis. Patients living in the most socioeconomically deprived areas of England are twenty percent more likely to have cancer diagnosed at a late stage as well as harbour more comorbidities than those in the least deprived areas [10]. These socioeconomic disparities per local area are captured in the UK by the Indices of Multiple Deprivation (IMD) (www.gov.uk/government/statistics/ english-indices-of-deprivation-2019). This uses census data to delineate residential postcodes into deciles from most to least deprived. Throughout treatment, patients from lower decile IMD postcodes are less likely to have definitive surgical management of malignancy, more likely to face substantial financial toxicity in attending appointments, and ultimately more likely to die of their disease [11–13].

Ethnicity is likewise recognised to play a key role in cancer outcomes and recruitment to clinical trials [7, 10]. This can be due to variations in profiles of disease, such as a high prevalence of triple-negative breast cancer in black compared to white females, or the high prevalence of melanoma skin cancer in white populations [14, 15]. It is recognised that minority ethnic groups are underrepresented in clinical trials across healthcare, as well as in cancer research [16, 17]. Further interlinking factors contributing to lifetime health determinants such as distance from centre, education, work environments and housing conditions as characterised by Dahlgren and Whitehead, additionally complicate patient journeys through cancer care and generate barriers to trials access [18, 19].

As treatments move towards a personalised oncology model, it is of increasing importance to consider the person with cancer holistically, including patient factors beyond tumour site and stage. A more complete understanding of the inequalities in enrolment to early phase trials will propagate a more fair and inclusive trial design for future cancer research.

## Summary of major findings

Social determinants of health play a significant role in referral and recruitment to early phase clinical trials in oncology in the North of England. When compared to expected referral population models, patients from the most deprived areas had a reduced likelihood of being referred for trials and patients from the most affluent an increased likelihood. Living beyond the median distance from Manchester reduced likelihood of consent to trials in Manchester ECMC. Data on ethnicity was not complete for both major centres. This identifies an area of unmet need to better characterise patient pathways and barriers to participation in early phase clinical trials in oncology to ensure equity of access and diversity of trial participants. This has important implications for equity of access and generalisability of trial results internationally and warrants further study.

## Materials and methods

Detailed clinicopathological outcome data for all patients referred to Newcastle and Manchester ECMCs for consideration of early phase clinical trials January 2021–December 2021 was collected. This was used to stratify consent to trial screening or pre-screening against SDoH characteristics, including: age, gender, IMD decile (1–10) as per residential postcode, ethnicity and distance to centre. Living beyond median referral distance to the centre was considered and longitudinal distances in miles between referred patients’ residential postcodes and respective ECMCs were calculated using an online distance calculating tool (www.freemaptools.com/how-far-is-it-between.htm).

Expected deprivation profiles of the respective referral populations in England were generated from online UK 2019 census data (www.imd-by-postcode.opendatacommunities.org/imd/2019). To generate representative deprivation profiles for each ECMC catchment area, residential postcodes of referred patients were used. For Newcastle ECMC, postcodes were used from the Northern Centre for Cancer Care (NCCC) referral areas (James Cook University Hospital, Carlisle and Freeman Hospital respectively). For Manchester ECMC, in initial data scoping, it was noted patient referrals were received from a wider geographic area. To limit demographic skew from regions with fewer referrals, only regions with five or more patients were included in the Manchester ECMC deprivation profile (Supplementary methods).

Patients referred from Scotland, Wales, Northern Ireland and the Republic of Ireland were excluded from the analysis due to incomparable or absent IMD data. Factors influencing non-enrolment to trial were explored thematically by IMD decile, yielding factors related to disease, treatment, and individual patient and administrative factors (Supplementary Table 1). IMD deciles were combined to quintiles for comparative and statistical analysis.

### Statistical analysis

Univariate analysis was utilised to determine odds ratios (OR) and 95% confidence intervals (CI) for respective determinants, *p* ≤ 0.05 were considered statistically significant. Complete analysis was performed separately for each centre, and comparisons of results were made between each centre to act as validators of individual ECMC findings.

### Ethics statement

This work was registered at each site respectively as a service evaluation audit. Permission was granted to collect retrospective patient data for this study by the respective Quality Improvement and Clinical Audit Committees (The Newcastle Upon Tyne Hospitals NHS Foundation Trust, audit number: 14040; The Christie NHS Foundation Trust, audit number: 3418).

## Results

### Demographics and social landscape of referrals

In 2021, 1243 patients with cancer were referred for consideration of early phase clinical trials to both Newcastle and Manchester ECMCs (*n* = 366, 29.4% and *n* = 877, 70.6% patients, respectively). Median age was 62 years (range: 18–90), and 670 (53.9%) were male. Age, sex, and primary tumour type distributions are represented in Table 1.

**Table 1:** Patients referred for consideration of early phase trials demographics Newcastle and Manchester ECMCs.

Characteristics	Newcastle *n* = 366 (%)	Manchester *n* = 877 (%)	Combined cohort *n* = 1243 (%)
Age (years)			
Median (range)	65 (23–90)	60 (18–87)	62 (18–90)
Gender			
Female	176 (48.1)	397 (45.3)	573 (46.1)
Male	190 (51.9)	480 (54.7)	670 (53.9)
Tumour type			
Colorectal	50 (13.7)	273 (31.1)	323 (26.0)
Lung	148 (40.4)	89 (10.1)	237 (19.1)
Prostate	24 (6.6)	66 (7.5)	90 (7.2)
Gynaecological	24 (6.6)	49 (5.6)	73 (5.9)
Breast	16 (4.4)	56 (6.4)	72 (5.8)
Pancreatic	9 (2.5)	45 (5.1)	54 (4.3)
Mesothelioma	31 (8.5)	19 (2.1)	50 (4.0)
Melanoma	8 (2.2)	29 (3.3)	37 (3.0)
Other	56 (15.3)	251 (28.6)	307 (24.7)

Lung cancer and mesothelioma were more prevalent in Newcastle ECMC referrals (Newcastle *n* = 148, 40.4% and *n* = 31, 8.5%; Manchester *n* = 89, 10.0% and *n* = 19, 2.1% respectively, *p* = <0.001, *p* = <0.0001) whereas colorectal cancer was more prevalent in the Manchester cohort (Newcastle *n* = 50, 13.7%; Manchester *n* = 273, 30.8%, *p* = <0.0001) (Table 2). Pancreatic cancer was also relatively over-represented in Manchester ECMC referrals compared with Newcastle ECMC (Newcastle *n* = 9, 2.5%; Manchester *n* = 45, 5.1%, *p* = 0.04) however, this was not statistically significant following Bonferroni correction.

**Table 2:** Referrals for prevalent tumour types to Newcastle and Manchester ECMCs.

Disease type	Newcastle referrals *n* = 366 (%)	Manchester referrals *n* = 877 (%)	Odds-ratio with 95% CI between ECMCs
Colorectal	**50 (13.7)**	**273 (31.1)**	**2.86, 2.05–3.98,** ***p*** < **0.0001**
Lung	**148 (40.4)**	**89 (10.1)**	**0.17, 0.12–0.22,** ***p*** < **0.0001**
Prostate	24 (6.6)	66 (7.5)	1.16, 0.71–1.88, *p* = 0.55
Gynaecological	24 (6.6)	49 (5.6)	1.19, 0.72–1.96, *p* = 0.51
Breast	16 (4.4)	56 (6.4)	1.49, 0.84–2.64, *p* = 0.17
Pancreatic	9 (2.5)	45 (5.1)	2.15, 1.04–4.44, *p* = 0.04
Mesothelioma	**31 (8.5)**	**19 (2.2)**	**0.24, 0.13–0.43,** ***p*** < **0.0001**
Melanoma	8 (2.2)	29 (3.3)	1.53, 0.69–3.38, *p* = 0.29

Statistically significant values are in bold.

Referred patients were majority White British (Newcastle *n* = 290, 79.2%, Manchester *n* = 451, 51.4%) (Table 3). This is in line with census data depicting a majority White British population in Northern England. Most remaining patients had ethnicity status as ‘other/unknown’ (Newcastle *n* = 64, 17.5%; Manchester *n* = 400, 45.6%). 364 of 366 (99.5%) patients in Newcastle ECMC had an English-speaking background. Spoken language data was not recorded for patients in Manchester ECMC.

**Table 3:** Referrals to Newcastle and Manchester ECMCs by ethnicity.

Recorded Ethnicity	Referred to Newcastle ECMC *n* = 366 (%)	Referred to Manchester ECMC *n* = 877 (%)	Combined ECMC cohort referrals *n* = 1243 (%)
White British	290 (79.2)	451 (51.4)	741 (59.6)
White Other	3 (0.8)	8 (0.9)	11 (0.9)
Chinese	3 (0.8)	2 (0.2)	5 (0.4)
Asian Other	2 (0.5)	1 (0.1)	3 (0.2)
White Irish	1 (0.3)	1 (0.1)	2 (0.2)
Asian or Asian British–Indian	1 (0.3)	6 (0.7)	7 (0.6)
Asian or Asian British–Pakistani	1 (0.3)	5 (0.6)	6 (0.5)
Black or Black British–Caribbean	1 (0.3)	3 (0.3)	4 (0.3)
Other/Unknown	64 (17.5)	400 (45.6)	464 (37.3)

**Fig. 1:**
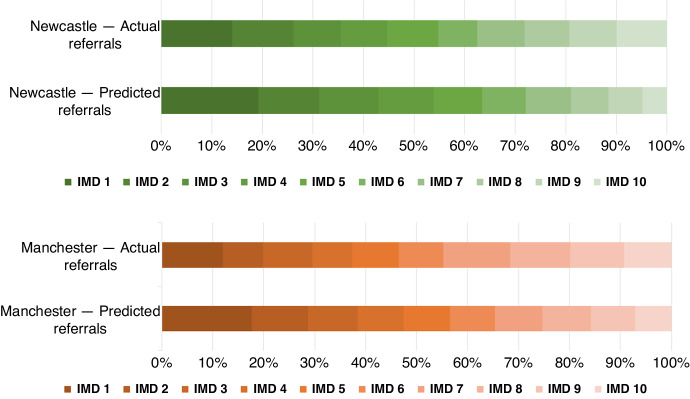
Distribution of IMD deciles in each ECMC deprivation profile compared with actual referred patients. Expected deprivation model for each ECMC referral catchment area (‘predicted referrals’ as a percentage), alongside actual patient referrals for each ECMC (‘actual referrals’ as a percentage). 75 of 1243 (6.0%) postcodes from combined ECMC referral cohorts were excluded from use in demographic modelling, including all patients living in Wales, Scotland, Northern Ireland, and English postcodes with lack of available IMD data as per recent census.

492,659 and 120,616 postcodes with associated IMD deciles were used to model the socioeconomic deprivation profiles of Manchester and Newcastle ECMC referral cohorts, respectively (Fig. 1).

### The influence of IMD decile on referrals

**Fig. 2:**
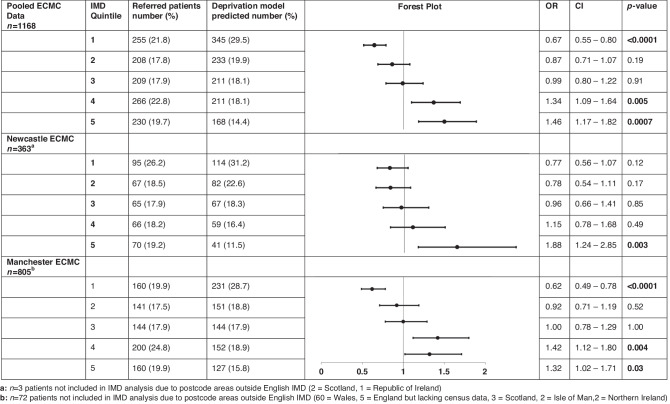
ECMC referrals per IMD Quintile. Odds ratios in forest plot depiciting the influence of patient IMD quintile on the odds of being referred to each ECMC compared with modelled ECMC demographic.

Combined ECMC data demonstrated in Northern England IMD quintile 1 patients (most deprived) had reduced likelihood of being referred for trials when compared to expected population models (OR, 0.67; 95% CI: 0.55 to 0.80, *p* = <0.0001), while IMD quintile 5 (least deprived) had increased likelihood of referral (OR, 1.46; 95% CI: 1.17 to 1.82, *p* = 0.0007) (Fig. 2).

In Manchester ECMC, patients resident in IMD quintile 1 were less likely to be referred for early phase clinical trials when compared to expected population models (OR, 0.62; 95% CI: 0.49 to 0.78, *p* = <0.0001). Patients resident in IMD quintile 5 had increased likelihood of referral (OR, 1.32; 95% CI: 1.02–1.71, *p* = 0.03).

In Newcastle ECMC, patients resident in IMD quintile 1 were less likely to be referred for early phase clinical trials when compared to expected population models, though this did not reach statistical significance (OR, 0.77; 95% CI: 0.56 to 1.07, *p* = 0.12). Patients from IMD quintile 5 were more likely to be referred (OR, 1.88; 95% CI: 1.24 to 2.85, *p* = 0.003).

### The influence of patient factors on being referred and enrolled

518 of 877 (59.1%) and 110 of 366 (30.1%) patients were consented to a study in Manchester and Newcastle ECMCs, respectively. Age, gender and IMD decile distributions were equivalent between patients referred and patients consented to studies across both ECMCs.

**Fig. 3:**
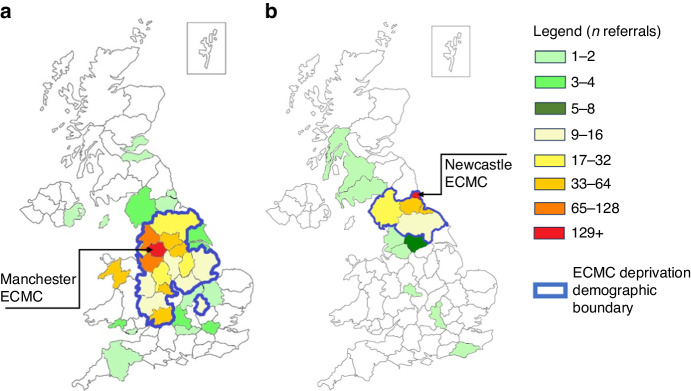
Postcode referral areas. Geographical heatmaps of UK postcode areas from which (**a**) Manchester ECMC and (**b**) Newcastle ECMC received referrals. n referrals represented as colours on heatmap. Outlined in blue denotes geographical areas in which respective ECMC deprivation profile postcodes were collected.

Distance to ECMC was collected for all patients and is displayed geographically (Fig. 3). Boundaries of included postcode areas as per methods section have been featured for reference. Referrals were received from all four nations of the UK and Republic of Ireland (ROI). In Manchester ECMC, the median distance to centre was 30.6 miles and living beyond this reduced the likelihood of being consented for early phase clinical trials (OR, 0.72; 95% CI: 0.55 to 0.94, *p* = 0.015). In Newcastle ECMC, the median distance to centre was 10.7 miles and living beyond this reduced the likelihood of being consented for early phase clinical trials but was not statistically significant (OR, 0.81; 95% CI: 0.52 to 1.27 *p* = 0.362).

Of those who were not consented to studies, 251 and 329 patients with IMD quintile data in Newcastle and Manchester ECMCs, respectively, were reviewed for factors related to non-consent (Supplementary Table 1). Factors were then compared between IMD quintiles 1 and 5. In combined ECMC data, disease, treatment, and patient and administrative factors did not vary significantly by IMD (Supplementary Table 2).

## Discussion

This work has demonstrated, for the first time, the inequalities of access to early phase clinical trials in the North of England. The influence of diversity and equity of access is increasingly being examined with respect to patient outcomes. In oncology, UK data has previously suggested that patients from higher socioeconomic backgrounds and of White British ethnicity are more likely to be referred for and recruited to early phase trials [7]. Furthermore, findings of sociodemographic inequalities have been demonstrated across early phase portfolios in other European countries, with the EGALICAN-2 study demonstrating inequalities in referral to and enrolment onto studies in France. This was demonstrated across comparable numbers nationally to our present large regional study [20]. In our work, a relative over representation of patients from affluent areas were represented in referrals, compared to predicted population models. In affluent areas, social determinants have been shown to have less adverse effects across the life course of patients and impact on their diagnosis with cancer [1]. Conversely, patients from more deprived areas were less likely to be referred. This may highlight recognised barriers to participation experienced by some patients with cancer including costs of travel, gaps in community support and later stage of diagnosis with less lead-time for referral [12]. Reduced referral rates may also be influenced by the recognised presence of greater comorbidities in more deprived socio-economic groups, which may be less compatible with early phase trials due to their stringent eligibility criteria [21].

Ethnicity in the Northern England cohorts demonstrated a White British propensity. This is characterised in the base population census data [22]. However, a significant proportion of patients had ethnicity ‘unknown’ (Newcastle *n* = 64, 17.5%; Manchester *n* = 400, 45.6%), which due to retrospective data collection was not possible to confirm. It is recognised that patients from minority ethnic backgrounds in the UK are less likely to participate in clinical research, as demonstrated through COVID-19 vaccine development, and improving access to studies is a National Institute for Healthcare Research (NIHR) priority [23–25]. Capturing ethnic diversity more comprehensively is of particular importance in early phase cancer trials due to the increasing number of molecularly guided interventional studies and the divergence in molecular target prevalence between ethnicities. An example of the relevance of this shift includes the increased incidence of EGFR mutations in Asian patients diagnosed with lung cancer [26].

Geographic distance from the ECMC was also demonstrated to be significant in reducing likelihood of consenting to an early phase trial once referred to the centre. This suggests that logistical and time restraints may impact on patients’ willingness to participate in trials, if residing in areas more remote from ECMCs. This was reflected in the findings of EGALICAN-2 [20]. Independent to this, the residual impact of the COVID-19 pandemic during 2021 may have had an impact on the willingness and ability of patients to travel greater distances for early phase trials, which was not investigated in this study.

Enrichment of specific tumour types was apparent in referral patterns, demonstrated by the relative increased representation of colorectal cancer in the Manchester cohort and thoracic malignancies in Newcastle. This may be indicative of disease-specific expertise in respective ECMCs. Of note, both primaries are more common in patients of lower socioeconomic status, which creates a greater imperative to promote access to trials in this socioeconomic group [27, 28]. Baseline demographic characteristics in the cohort were similar to previous published cohorts [12, 20] with male predominance noted. A higher than average referral age was noted particularly within the Newcastle cohort (median age 65 years; EGALICAN-2 62 years; Noor et al. (London) 60 years). Increasing age is generally perceived to be a barrier to participation in trials and confounds generalisability of study results to older populations which is important to consider in expanding the diversity of early phase trial patients [29]. This study demonstrates a positive diversity of age in some UK centres in consideration for enrolment to trials, in the context of an ageing and increasingly multimorbid population with cancer.

Reasons for non-enrolment to early phase clinical trials were categorised as Patient, Disease or Treatment factors. There was no significant difference for non-enrolment reason categories, as described in Supplementary Table 1, between IMD quintiles 1 and 5. While a detailed analysis of this remains beyond the scope of this manuscript, this exploratory analysis highlights the need for future research to better understand barriers to participation in clinical trials both before and after referral to an ECMC.

This study has limitations in that the deprivation profiles for each ECMC were based on census area population data as opposed to same centre matched cohorts of patients with cancer. The catchment area of Manchester ECMC was larger than that of Newcastle and received large numbers of distant referrals. These included 65 patients from other devolved nations without equitable IMD data. The skew in geographic contribution from distant referrals in England was accounted for by excluding areas with <5 patients referred. This created a robust population model for which to generate expected referral numbers which could not be replicated by local centre matched cohorts alone. Furthermore, the retrospective nature of data collection highlighted disparities in documentation, particularly that of patient ethnicity. In recognising that this is important to evaluate services in both ECMCs in equality of access, more emphasis should be placed on capturing patient ethnicity at the time of first presentation to ECMCs.

## Conclusions

Inequalities in socioeconomic and geographic factors as determinants of health have significant influence in referral and enrolment to early phase clinical trials in Northern England. We have identified an area of unmet need which warrants further characterisation and intervention to increase referral and recruitment of patients of lower socioeconomic status.

Future work should involve prospective national socio-demographic characterisation of patients in early phase clinical trials in oncology and increased understanding of barriers to participation. This study has important implications for equity of access and generalisability of early phase trial results internationally.

### Supplementary information

Supplementary Tables

Supplementary Methods

## Data Availability

Data is available on reasonable request from the corresponding author.
