# Supplementary material for: Social determinants of health inequalities in early phase clinical trials in Northern England
Source: Br J Cancer. 2024 Jun 24;131(4):685–91. doi: 10.1038/s41416-024-02765-w (PMC11333496; doi:10.1038/s41416-024-02765-w)
Supplement: Supplementary file 1 — Supplementary Tables [file 41416_2024_2765_MOESM1_ESM.docx]

**SUPPLEMENTARY DATA**

***Supplementary Table 1.*** *Categorisation of factors for non-enrolment were collected by manual review of clinical records and derived into the thematic categories.*

| **Disease Factors** | - Progression / deterioration of health - Performance status exclusion - Exclusion criteria (other) - brain metastases, pathological fracture, blood tests, co-morbidities - Death - No active cancer - Disease could not be biopsied for trial considered - No trial options available - No measurable disease - Patient remained on waiting list at data closure |
| --- | --- |
| **Treatment Factors** | - Treatment available outside of trial - Late phase trial available - Trial at other ECMC - Compassionate access to therapeutics - Referred for genomic sequencing only |
| **Patient and Administrative Factors** | - Patient decision - Lost to follow up - Did not attend appointment |

***Supplementary Table 2.*** *Reasons for not being consented to a clinical trial displayed for IMD Quintile 1 and Quintile 5 patient referrals, for both ECMC centres, divided into disease, treatment, and patient factors.*

| **Reasons not on trial** | **Quintile 1 n = 126 (%)** | **Quintile 5 n = 107 (%)** | **P-value** |
| --- | --- | --- | --- |
| **Disease Factor** | 62 (49.2) | 58 (54.2) | 0.4467 |
| **Treatment Factor** | 36 (28.6) | 33 (30.8) | 0.7053 |
| **Patient Factor** | 28 (22.2) | 16 (15.0) | 0.1577 |
