# Supplementary material for: Social determinants of health inequalities in early phase clinical trials in Northern England
Source: Br J Cancer. 2024 Jun 24;131(4):685–91. doi: 10.1038/s41416-024-02765-w (PMC11333496; doi:10.1038/s41416-024-02765-w)
Supplement: Supplementary file 2 — Supplementary Methods [file 41416_2024_2765_MOESM2_ESM.docx]

**SUPPLEMENTARY METHODS**

Generation of ECMC deprivation demographic

ECMC demographics were generated using postcode data for all referred patients. Major patient postcodes were grouped by locality by removing all characters other than the stem, e.g. using the sample postcode ‘AB12 CD34’, characters beyond the stem would be removed, leaving ‘AB’, to correspond to the postcode area. Greater variety of postcode referral areas was observed in Manchester than may be expected from the Christie NHS Foundation Trust’s referral area in isolation, therefore postcode areas in which 5 or more patients were referred from to Manchester ECMC were included in the Manchester deprivation profile to better represent the deprivation areas of patients referred. The Newcastle ECMC referral area for the Northern Centre for Cancer Care (NCCC) referral region (James Cook University Hospital, Carlisle and Freeman Hospital respectively) was well represented in the patients referred therefore this expansion of geographical boundaries was not applied so as to not introduce skew to the deprivation profile represented.

For each major postcode area all active and inactive postcodes were identified using an online generator (www.doogal.co.uk/UKPostcodes?Search). IMD deciles were identified for all postcode areas within the deprivation profile using the government IMD search engine. This generated what the expected prevalence of each IMD decile within the general population for each ECMC would be.

Postcodes for actual patients referred were then linked to corresponding IMD deciles. This generated a deprivation profile of actual referrals by percentages of IMD deciles which could be compared to expected population models. For analysis these were combined into quintiles as is established in the published literature.
